# An Uncommon Presentation of Unstable Ventricular Tachycardia: Raising Awareness for Early Recognition of Chagas Disease

**DOI:** 10.7759/cureus.61189

**Published:** 2024-05-27

**Authors:** Saimanoj Guntaka, Allan Lin, Suhwoo Bae, Michael Vaysblat, Matthew Pierce

**Affiliations:** 1 Internal Medicine, Northwell Health, Manhasset, USA; 2 Cardiology, Northwell Health, Manhasset, USA

**Keywords:** sudden cardiac death (scd), implantable-cardioverter defibrillator, trypanosoma cruzi, ventricular tachycardia (vt), chagas cardiomyopathy

## Abstract

Chagas disease (CD), caused by *Trypanosoma cruzi,* is a leading cause of cardiomyopathy in Latin America that can lead to heart failure, arrhythmias, and sudden cardiac death (SCD). We present a case of a 71-year-old female from El Salvador with symptomatic ventricular tachycardia (VT) requiring emergent cardioversion and implantable cardioverter-defibrillator (ICD) due to CD. Diagnostic evaluation is limited and unclear in cases of chronic disease. Treatment involves antiparasitic therapy, heart failure management, and arrhythmia prevention. With growing numbers of cases in the US and limited treatment options, we highlight the need for timely recognition and intervention to reduce the burden of CD.

## Introduction

Chagas disease (CD), caused by the protozoan *Trypanosoma cruzi,* is a leading cause of non-ischemic cardiomyopathy in Latin America [1,2]. Once thought of as a tropical disease, a growing number of cases in the US combined with a lack of recognition is leading to progressive heart failure, arrhythmias, and sudden cardiac death (SCD) in immigrants from endemic areas [2,3]. With increasing prevalence largely due to immigration and globalization, especially in the West and South regions of the US, strong suspicion should be raised in these individuals to prevent SCD [3]. We present a case of a patient from El Salvador with symptomatic ventricular tachycardia (VT) in the setting of CD who received an implantable cardioverter-defibrillator (ICD) for secondary prevention of SCD.

## Case presentation

**Figure 1:**
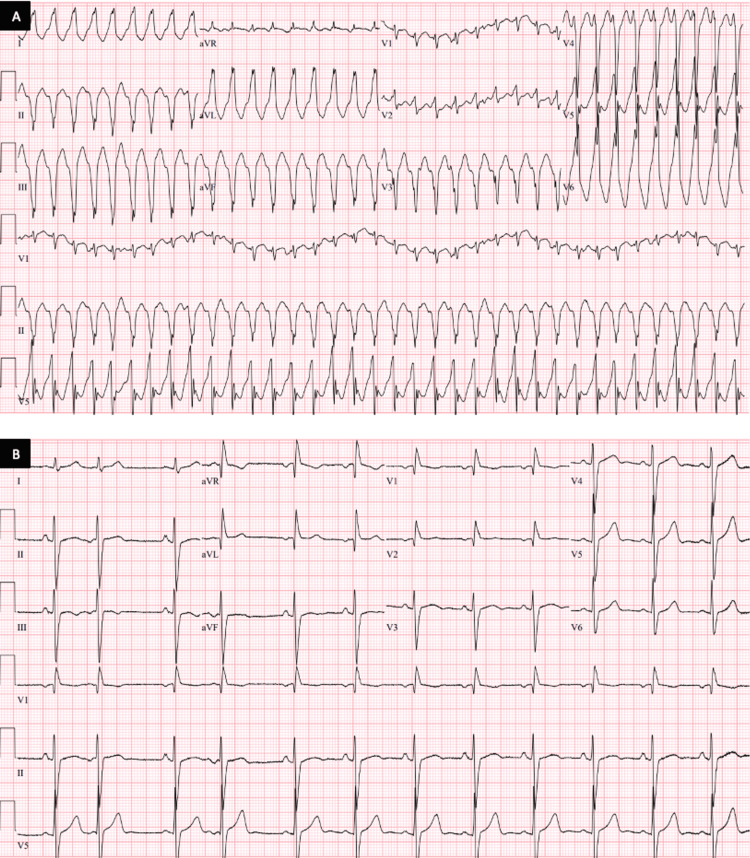
Electrocardiogram (A) Admission EKG showing monomorphic VT at over 200 beats per minute, and (B) EKG during the hospitalization course showed persistent premature atrial complexes along with a right bundle branch block. VT: ventricular tachycardia

A 71-year-old female from El Salvador with no significant medical history was brought to the emergency room by her daughter after being found unconscious on the floor. Upon arrival, she complained about substernal, radiating chest pain, associated with multiple syncopal episodes over the past few weeks. Her initial vitals revealed unstable VT at 217 beats per minute (Figure 1) and a blood pressure of 83/42 requiring emergent cardioversion. Subsequent bloodwork revealed a lactate of 2.5 mmol/L with electrolytes and renal function within normal limits. Her high-sensitivity cardiac troponin was 57 ng/L. Pro-brain natriuretic peptide (Pro-BNP) was 5936 pg/mL. Chest X-ray revealed clear lungs with no cardiomegaly.

**Figure 2:**
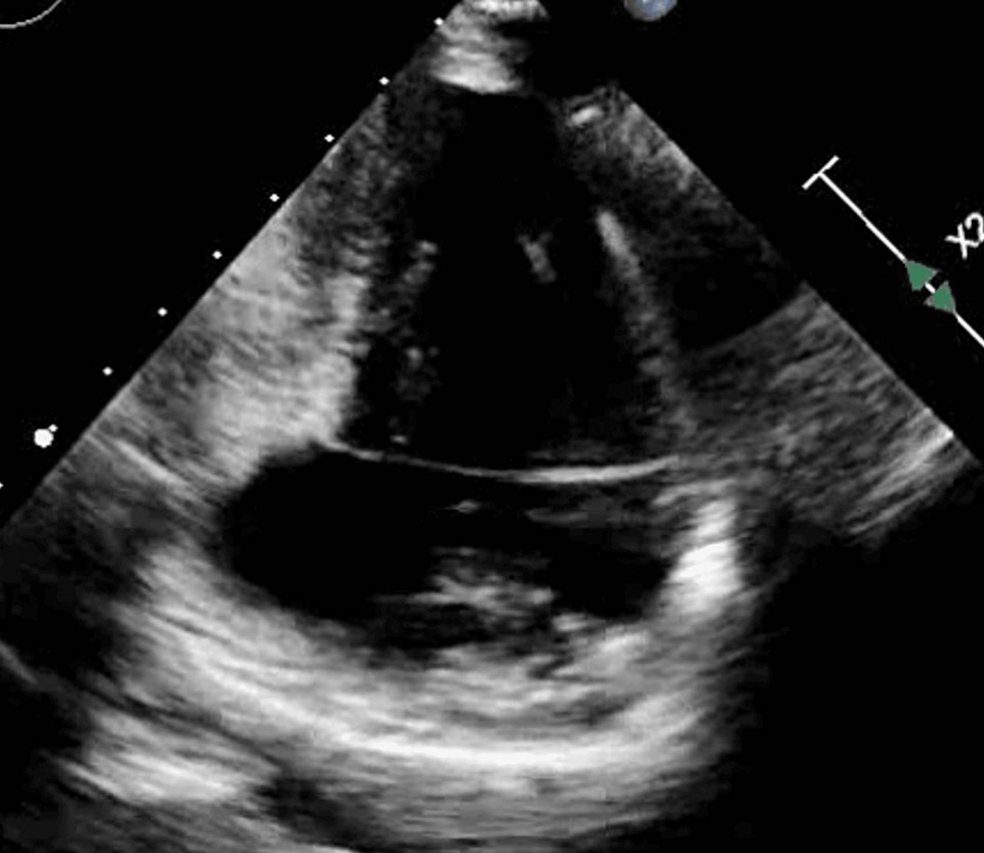
TTE TTE demonstrating basal inferior wall aneurysm in the apical two-chamber view. TTE: transthoracic echocardiogram

**Figure 3:**
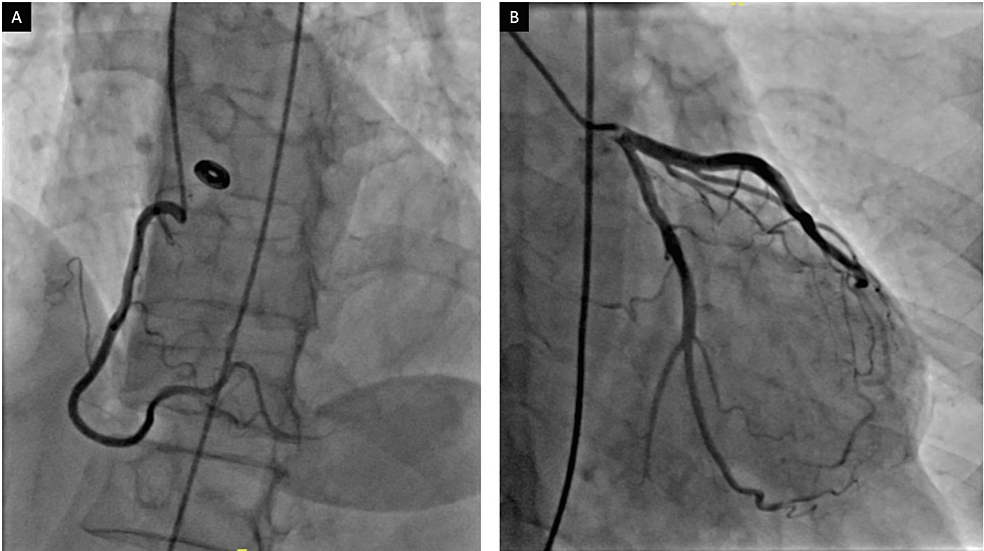
Cardiac angiography (A) Right and (B) left coronary arteries with non-obstructing coronary disease.

**Figure 4:**
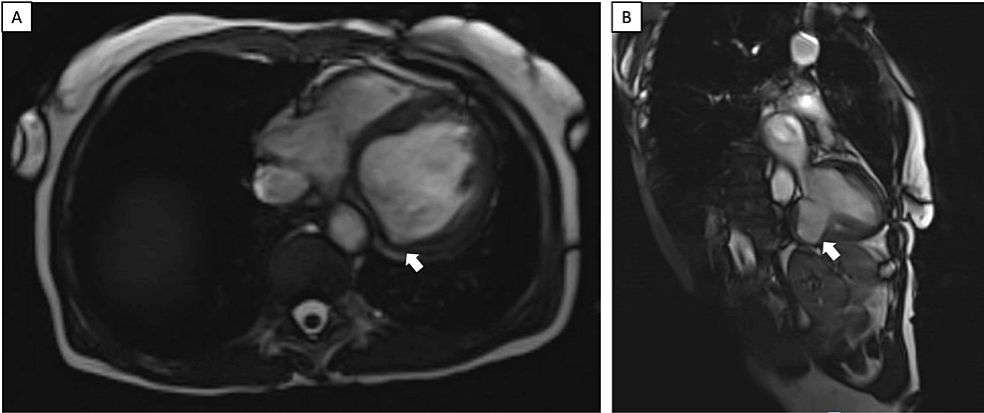
CMR Aneurysmal dilatation (white arrows) of the basal inferior and inferolateral left ventricular wall in (A) transverse and (B) sagittal views. CMR: cardiovascular magnetic resonance

**Figure 5:**
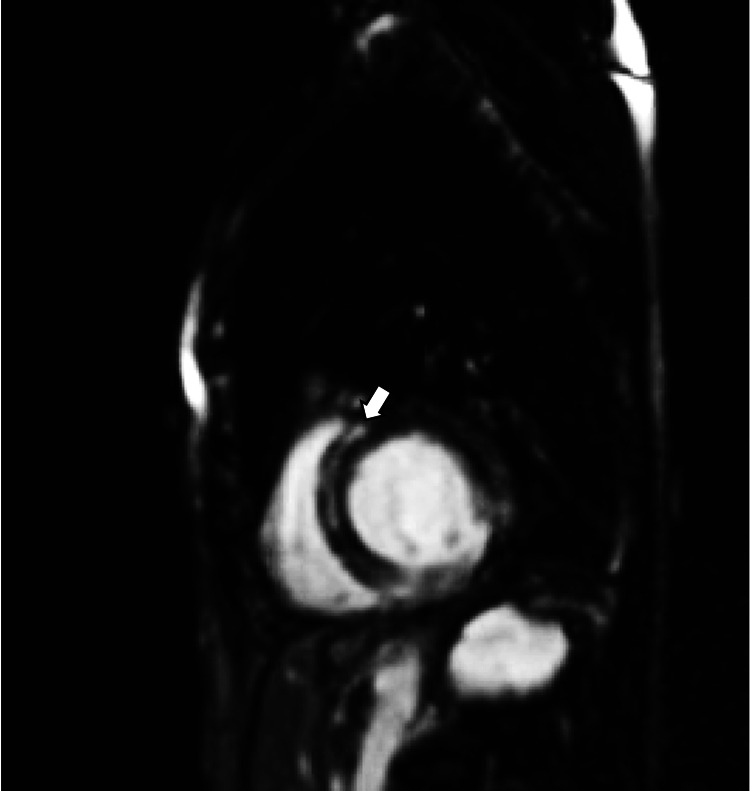
CMR with contrast LGE is shown with a white arrow on a CMR short-axis view. LGE was present in multiple mid-myocardial segments. LGE: late gadolinium enhancement; CMR: cardiovascular magnetic resonance

A transthoracic echocardiogram (TTE) was performed showing a reduced left ventricular (LV) ejection fraction of 25% with severe global systolic dysfunction, basal inferior wall aneurysm, and moderate diastolic dysfunction (Figure 2). She underwent a left heart catheterization, which revealed non-obstructive coronary disease (Figure 3). A Cardiovascular magnetic resonance (CMR) performed confirmed impaired systolic function with aneurysmal dilatation of the basal inferior and inferolateral LV walls (Figure 4). Late gadolinium enhancement (LGE) showed mid-myocardial enhancement in the basal to mid anteroseptal, inferoseptal, anterolateral, and inferolateral segments (Figure 5).

With a history of immigration two months ago from El Salvador and recurrent episodes of syncope, there was a strong suspicion of Chagas cardiomyopathy as a plausible explanation. She underwent serologic testing for *T. cruzi* IgG antibody confirming a diagnosis of Chagas cardiomyopathy. With no Food and Drug Administration (FDA) approved medications for CD in adult patients along with ineffective treatment for chronic CD, treatment for CD was not pursued. She underwent electrophysiology studies with subsequent endocardial ablation of an inducible VT located within and at the borders of the inferolateral aneurysm. No sustained arrhythmias were induced with programmed stimulation following the ablation. Further EKGs performed during hospitalization revealed persistent and intermittent atrial arrhythmias without sustained ventricular rhythms (Figure 1). An ICD was placed for secondary prevention of SCD, and she was started on guideline-directed medical therapy (GDMT) with losartan, spironolactone, and metoprolol succinate. She now follows closely with her primary care physician, cardiologist, and electrophysiologist. To date, there have been no further hospitalizations and no sustained arrhythmias documented from ICD reports. She is also currently scheduled to visit gastroenterology for further evaluation of the gastrointestinal system with an upper endoscopy.

## Discussion

CD is a leading cause of non-ischemic cardiomyopathy in Latin America. Although endemic to Central and South America, CD is presenting as a significant public health concern due to the growing number of immigrants in the US [2]. Transmission of *T. cruzi* occurs through the triatome bug, found in rural areas with poor conditions. The protozoan enters the host’s bloodstream through breaches in the skin and causes a systemic infection [2]. Less frequently, transmission can also occur through blood transfusions, vertically from mother to child, and rarely, via oral transmission [1].

CD is a progressive disease with an acute phase, an indeterminant phase, and a chronic phase. The acute phase of CD typically lasts about one to two weeks. While many patients may not exhibit symptoms, some may experience fever, fatigue, and weakness. Progression to the indeterminant phase follows an asymptomatic course of variable length. The chronic phase of the disease presents with a variety of symptoms including shortness of breath, chest pain, palpitations, and syncope. Chagas cardiomyopathy is a complication of the chronic phase, leading to heart failure, arrhythmias, and SCD [2,4,5].

The pathogenesis of CD is thought to be multifactorial, primarily involving the presence of the protozoan in the bloodstream and soft tissues. Several hypotheses have been developed to help understand the mechanism for cardiac dysfunction. Direct tissue damage from parasitemia can lead to an inflammatory response that, if prolonged, exacerbates cardiac damage [5,6]. Microvascular damage has been implicated in microinfarcts and endothelial dysfunction leading to segmental wall motion abnormalities (WMA), myocardial thinning, and ventricular aneurysms [4,6]. Autonomic dysfunction from denervation can also occur, with studies demonstrating the involvement of both parasympathetic and sympathetic pathways [6-8]. It is speculated that a possible correlation exists between the severity of ventricular arrhythmias and the degree of autonomic denervation [7,8]. Chagas cardiomyopathy has a worse prognosis and higher mortality rates compared to other forms of dilated cardiomyopathies [4,6]. Apical lesions and aneurysms in the LV are distinct features of Chagas's heart disease, contributing to systolic dysfunction and predicting complications such as mural thrombus formation and stroke [6,9]. Complex arrhythmias can occur as well, including sinus node dysfunction, right bundle branch block, atrial fibrillation, AV blocks, and ventricular arrhythmias [4].

The diagnostic imaging modality of choice is a TTE, which can detect segmental WMAs, most frequently located within the LV. Progression of the disease can lead to the formation of aneurysms within the LV. A CMR can further quantify myocardial edema and scar formation with LGE to help determine pump function and risk for SCD [4]. Notably, these intramural scars serve as the arrhythmogenic substrate for endocardial and epicardial ventricular circuits, with up to 70% of patients experiencing VT from the inferolateral LV and, less frequently, LV apex [4]. Epicardial circuits are more common, however, are harder to ablate due to procedural and anatomical difficulties [4].

Treatment of CD with benznidazole or nifurtimox is indicated in the acute phase with a cure rate of 60-80% [2,5]. However, indications for treatment in the chronic phase remain unclear with persistent positive serology despite treatment. Furthermore, complications of chronic CD such as Chagas cardiomyopathy have no proven treatment. Treatment for heart failure mirrors that of other non-ischemic cardiomyopathies, although limited data exist specifically for CD. Treatment for arrhythmias reflects standard guidelines including VT ablation via endocardial and epicardial approach. Similar to other forms of cardiomyopathy, an ICD for primary and secondary prevention is also indicated, albeit lacking strong evidence specific to CD patients [4,5].

## Conclusions

We present an increasingly common case of chronic CD leading to Chagas cardiomyopathy with resultant arrhythmias. Our case highlights the importance of considering CD as a potential etiology in patients from endemic regions who present with heart failure or complex arrhythmias. Moving forward, increased awareness, early detection, and access to appropriate therapies are essential in mitigating the burden of CD-related cardiovascular disease.
